# 3D Culture of MIN-6 Cells on Decellularized Pancreatic Scaffold: In Vitro and In Vivo Study

**DOI:** 10.1155/2015/432645

**Published:** 2015-11-24

**Authors:** Di Wu, Jian Wan, Yan Huang, Yibing Guo, Tianxin Xu, Mingyan Zhu, Xiangjun Fan, Shajun Zhu, Changchun Ling, Xiaohong Li, Jingjing Lu, Hui Zhu, Pengcheng Zhou, Yuhua Lu, Zhiwei Wang

**Affiliations:** ^1^Department of General Surgery, Affiliated Hospital of Nantong University, Nantong, Jiangsu 226001, China; ^2^Surgical Comprehensive Laboratory, Affiliated Hospital of Nantong University, Nantong, Jiangsu 226001, China

## Abstract

Type 1 diabetes is an autoimmune disease which is due to the lack of *β* cells. The ideal therapy to cure the disease is pancreas transplantation, but its application is confined to a limited number of people due to the shortage of organ and the need for life-long immunosuppression. Regenerative medicine methods such as a tissue engineered pancreas seem to provide a useful method. In order to construct a microenvironment similar to the native pancreas that is suitable for not only cell growth but also cellular function exertion, a decellularized mouse pancreas was used as a natural 3D scaffold in this experiment. MIN-6 *β* cells were planted in the bioscaffold. The cell engraftment was verified by HE staining and SEM. Immunostaining procedures were performed to confirm the normal function of the engrafted cells. qRT-PCR demonstrated that insulin gene expression of the recellularized pancreas was upregulated compared with conventional plate-cultured cells. In vivo experiment was also accomplished to further evaluate the function of the recellularized bioscaffold and the result was inspiring. And beyond doubt this will bring new hope for type 1 diabetic patients.

## 1. Introduction

Diabetes is a global disease which has raised great economic and social problems. Nearly 285 million people around the world suffer from diabetes [1]. Type 1 diabetes mellitus is characterized by high blood glucose level caused by the lack of insulin-secreting cells (*β* cells) [2]. The treatment of diabetes mellitus remains inadequate. Currently, the treatment of diabetes is focused on the change of life-long lifestyle, pharmaceutical intervention, and insulin supplementation, but less than 40% of patients have cured the disease [3]. Also, these methods do not reduce the long term complications caused by diabetes mellitus [4]. *β* cell replacement, by either pancreas or islet cell transplantation, is the only treatment to establish long term, stable euglycemia in the diabetic cases [5] and has been proved to improve quality of life [6] and reduce secondary complications compared to insulin treatment [7–9]. However, clinical application of the transplantation process has been hindered by the following limitation: the shortage of donor and the need for immunosuppression in the whole life [5]. In recent years, the production of insulin-secreting cells originated from isogenous cells has achieved great improvements, which seems to have solved the problems mentioned above. However, the gradual decrease of cell function and activity after transplantation hindered the use of transplantation of insulin-secreting cell in the treatment of type 1 diabetes mellitus [10].

Development in tissue engineering has prompted the advance of replacement of tissues or organs [11, 12]. Ideally, a bioscaffold should provide the same microenvironment niche to the cells grafted as that of a native ECM. Successful fabrication of ECM scaffolds and recellularization of ECM scaffolds have been reported in many organs [13–19]. Right now, the pancreatic tissue engineering is still working on creating a microenvironment similar to the native pancreas [20]. We believe that decellularized pancreatic scaffolds simulate the microenvironment at the greatest extent. Very few studies up to now have successfully manufactured the decellularized pancreatic ECM and repopulated it. A previous study has described the successful fabrication and recellularization of a decellularized pancreatic bioscaffold [21]. The result was inspiring and promoted tissue engineering of the pancreas to a large extent.

In this study, we introduced freeze-thaw cycle which can dissolve cells in tissue and organ effectively and thus facilitate the later decellularization protocol, while having minor influence on ultramicrostructure of the tissue or organ [22]. Besides, freezing and thawing cycle can reduce immunogenicity of the bioscaffold such as leukocyte infiltration [23]. Also, in our study, we can see clearly that cells were well distributed around the ECM. Furthermore, in vivo studies were also done to further evaluate the histocompatibility and the function of the recellularized scaffolds.

## 2. Materials and Methods

The cell line MIN-6 *β* was bought from Type Culture Collection of the Chinese Academy of Sciences (Shanghai, China). C57BL/6J mice were from the animal center of Nantong University. All animal work was according to the Animal Welfare Act and was approved by the Animal Ethics Committee of the Medical School of Nantong University.

### 2.1. Decellularization of the Harvested Mouse Pancreas

Male C57BL/6J mouse, weighing approximately 28 g, was anesthetized with chloral hydrate. A celiotomy was performed and a detaining needle was inserted into the portal vein of the mouse and ligated in place to allow retrograde perfusion. The superior mesenteric vein, splenic vessels, and the vessels between the pancreas and the small intestine were ligated. Then the pancreas was separated from all adjacent organs and tissues. The harvested pancreas was connected to a peristaltic pump (MasterFlex L/S) to allow perfusate to flow through the hepatic portal vein at the speed of 4 mL/min. First of all, double distilled water was used to wash out the remaining blood in the vessel. Then the pancreas was stored at −80°C for one day and thawed at room temperature. After that, the pancreas was perfused with PBS which contains 1% Triton X-100 (Sigma Aldrich)/0.1% ammonium hydroxide. After the tissues became translucent (about 4 h), the remaining detergent was cleared from the pancreas by perfusion with 1 L PBS.

### 2.2. Angiography of the Intact Vasculature

After decellularization, a kind of blue dye was infused through the detaining needle to see if the vasculature was kept intact which is crucial for the later recellularization protocol.

### 2.3. Histological Evaluation of the Bioscaffold

For evaluation of the effectiveness of our decellularization process, hematoxylin-eosin (HE) staining technique was conducted. The decellularized scaffolds were fixed in formalin for more than 24 h and embedded in paraffin and the thickness of the sections was 5 *μ*m. And then they were moved to 60% ethanol. After that, they were dehydrated in alcohols, immersed in chloroform, and embedded in paraffin wax. Sections were fixed on slides and stained with hematoxylin and eosin.

### 2.4. Testing of Biocompatibility

For the study of biocompatibility, a 1 cm^2^ pancreatic scaffold was sutured subcutaneously in back of male C57BL/6 mice. Briefly, the mouse was anesthetized with chloral hydrate and surgical site was sterilized by 75% ethanol. An incision measuring approximately 1.5 cm was made on the dorsal side. The decellularized pancreatic scaffold was fixed to the subcutaneous tissue with four 5-0 prolene sutures and the incision was closed layer by layer. The incision place was disinfected every other day using 75% ethanol. Following execution the pancreatic scaffold and the surrounding tissue were harvested at 3 d, 7 d, and 14 d, respectively, and fixed in formalin for hematoxylin-eosin (HE) staining.

### 2.5. Immunofluorescence Staining of ECM Component

Sections of the native and decellularized pancreas were stained in the following protocols. Sections were blocked with 5% BSA for 30 min at 25°C and then were incubated with primary antibody (Anti-Collagen I antibody, 1/200 in PBS, Abcam) for 12 h at 4°C. Goat Anti-Rabbit IgG H&L (Alexa Fluor 488, Abcam) diluted at a concentration of 1 : 400 was used as the secondary antibody.

### 2.6. Scanning Electron Microscopy (SEM) Analysis

Samples taken from the scaffold were fixed in glutaraldehyde (Sigma) overnight and fixed with 1% osmic acid and then were dehydrated in 30%, 50%, 70%, and 90% alcohols successively and put in a critical point dryer for 10 min. Specimens were overgilt to obtain electric conductivity. Samples were visualized by a scanning electron microscope.

### 2.7. DNA Assessment

Total DNA in the native and decellularized pancreata (*n* = 3) were extracted with QIAamp DNA Mini Kit (Qiagen). The concentration of the DNA in the extracting solution was measured by nanophotometer (IMPLEN).

### 2.8. Recellularization of Decellularized Pancreas

MIN-6 cells were cultured in Dulbecco's modified Eagle's medium (Corning) containing 10% FBS (Gibco), 50 *μ*M beta-ME, and 100 U/mL penicillin/streptomycin (Corning), at 37°C and 5% CO_2_ atmosphere. Before recellularization, Co 60 irradiation was used to sterilize the decellularized pancreatic scaffold. The system was placed in a standard CO_2_ (5%) cell incubator at constant temperature (37°C). MIN-6 cells (30 × 10^6^) were trypsinized and suspended in 1 mL culture medium. They were infused into the decellularized pancreas via the detaining needle in 3 steps, 0.3 mL each, and between each step, there was a 20 min interval. After that, the scaffold was left undisturbed to allow the engrafted cells to attach for 2 h. Then the recellularized scaffold was continuously perfused through the portal veins (PVs) at the speed of 2 mL/min. The speed was controlled by peristaltic pump. The medium needed to be changed every day. After 5 days the scaffold was taken out.

### 2.9. Histological Examination of the Recellularized Pancreas

The recellularized scaffolds were fixed in formalin for more than 24 h and embedded in paraffin and the thickness of the sections was 5 *μ*m. And then they were moved to 60% ethanol. After that, they were dehydrated in alcohols, immersed in chloroform, and embedded in paraffin wax. Sample was sectioned onto object slide. Sections were at last stained with hematoxylin and eosin.

### 2.10. Immunostaining Procedures

We assessed the functional characteristics of engrafted MIN-6 cells in the decellularized matrix via immunostaining at 5 d of culture of insulin whose presence indicated MIN-6 cells viability and function. For immunostaining, sections went through antigen retrieval. Sections were incubated with primary antibodies diluted in PBS which contained 2% (v/v) bovine serum albumin. The following primary antibody was used: guinea pig anti-insulin antibody (Abcam), and the secondary antibody was HRP AffiniPure Goat Anti-Guinea Pig IgG (H + L).

### 2.11. qRT-PCR

RNA from cells cultured on the culture dish and the recellularized scaffold was extracted by Qiagen 74104 RNeasy Mini Kit following the manufacturer's recommendation. The absorbance of the sample was measured by nanophotometer (IMPLEN) to obtain RNA concentration and quality. 1 mg of RNA was reversely transcribed into cDNA with the RevertAid First Strand cDNA Synthesis Kit (Fermentas, Thermo Fisher Scientific, Waltham, MA, USA) following the manufacturer's recommendation. Primers were designed as our previous study [24]. Ins1 and ins2 were used as insulin markers and qRT-PCR was executed, and it was repeated three times.

### 2.12. Establishment of Diabetic Model in Mice

30 C57BL/6J mice were divided into 6 groups (*n* = 5): I: healthy mice; II: diabetic mice without interference; III: diabetic mice with subcapsular injection of the liver of MIN-6 *β* (cell amount: 2 × 10^5^); IV: diabetic mice with scaffold implantation group; V: diabetic mice with recellularized scaffold implantation group (cell amount: 2 × 10^5^); VI: diabetic mice with recellularized scaffold cubes implantation group (bioscaffolds were chopped into 0.5 × 0.5 cm^2^ and cocultured in the 96-well plate with MIN-6 cell suspension (cell amount: 2 × 10^5^)). STZ were intraperitoneally injected at 160 mg/kg based on the previous literature [25], and group I was not injected. The mice with fasting glucose higher than 20 mM were considered as successful models.

### 2.13. In Vivo Implantation in Diabetic Mice

3% pentobarbital sodium was intraperitoneally injected to anesthetize the mice before transplantation. In group III, mice were injected with 1 mL of cell suspension to the subcapsule of the hepatic lobe. In groups IV, V, and VI, the scaffolds and the recellularized scaffolds were implanted subcutaneously by suturing to the adjacent tissue. After surgery, the fasting glucose was measured every day in the first three days and then every three days.

### 2.14. Statistical Processing

Data of each group was repeated 3 times. And data were expressed as mean ± SD. SPSS 19.0 was used for One-Way-ANOVA, with *P* < 0.05 considered as statistically significant difference.

## 3. Results

### 3.1. Preparation and Evaluation of the Decellularized Pancreatic Scaffold

**Figure 1:**
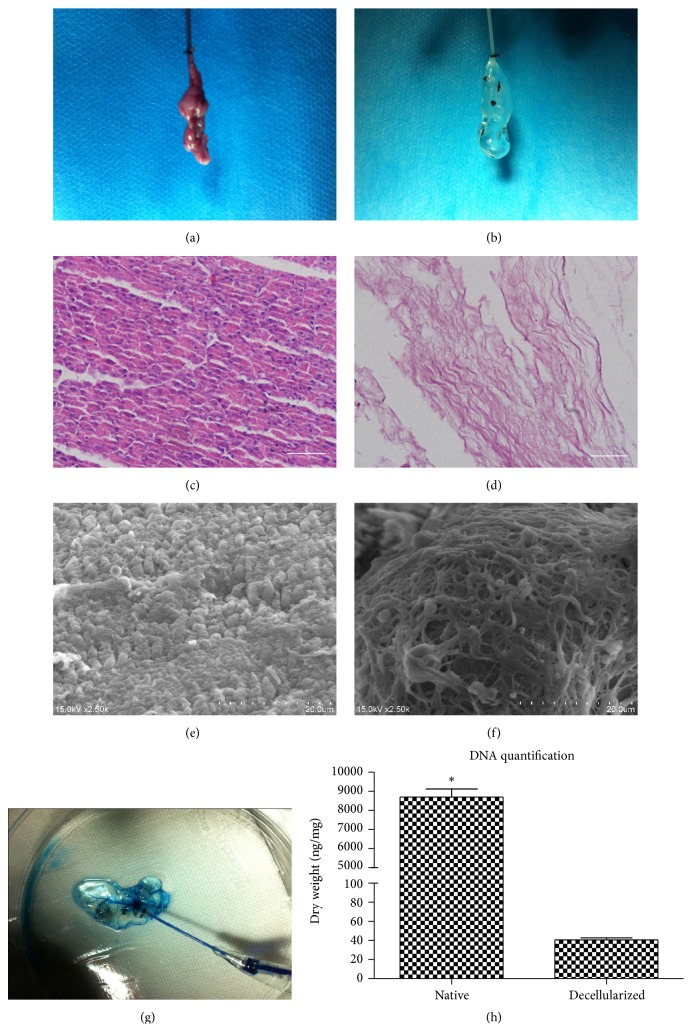
Perfusion-decellularization of murine pancreas. Gross picture: (a) native pancreas, (b) decellularized pancreas. HE staining: (c) native pancreas, (d) decellularized pancreas. Bar = 100 *μ*m. SEM analysis: (e) native pancreas, (f) decellularized pancreas. (g) Angiography affirmed the intact vasculature branches. (h) DNA quantification. ^*∗*^ *P* < 0.05.

After perfusion with detergent solution, the color of the mouse pancreata changed from red to translucent (Figures 1(a) and 1(b)). The cellular material of the native pancreas (Figure 1(c)) was removed and the decellularized pancreatic matrix demonstrated no nucleus left (Figure 1(d)). SEM confirmed a lot of cellular material of the native pancreas (Figure 1(e)) and the removal of cellular material and preservation of collagen fibers (Figure 1(f)). The result of DNA quantification showed that the amount of DNA decreased from 8696.3 ± 427.3 ng/mg in native pancreas to 40.7 ± 2.1 ng/mg in decellularized pancreatic scaffold (*P* < 0.05) (Figure 1(h)). After the infusion of trypan blue dye, the intact vasculature branches can be seen (Figure 1(g)).

### 3.2. ECM Characterization: Immunofluorescence Staining

**Figure 2:**
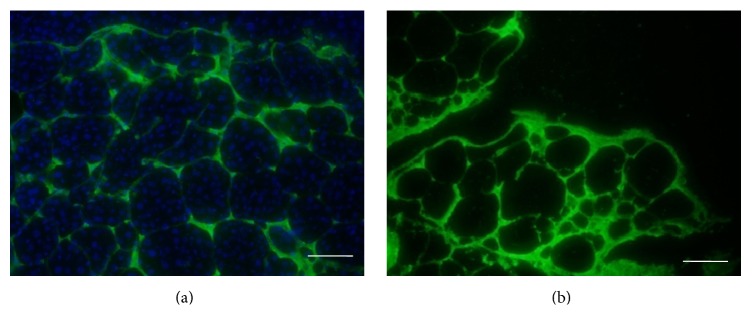
Immunofluorescence staining of collagen I and nucleus: (a) native pancreas, (b) decellularized pancreas. Bar = 100 *μ*m.

Immunofluorescence staining of ECM component showed the preservation of collagen I after decellularization process. After decellularization, collagen I was preserved, and the DAPI staining (Figure 2(b)) demonstrated no cellular material left compared to the native pancreas (Figure 2(a)). This finding suggests that our decellularization method completely removes all of the cellular elements and preserves the ECM composition at the same time. Besides, the ECM structure of the decellularized pancreatic scaffold looked like that of the native pancreas.

### 3.3. In Vivo Response to Decellularized Pancreas

**Figure 3:**
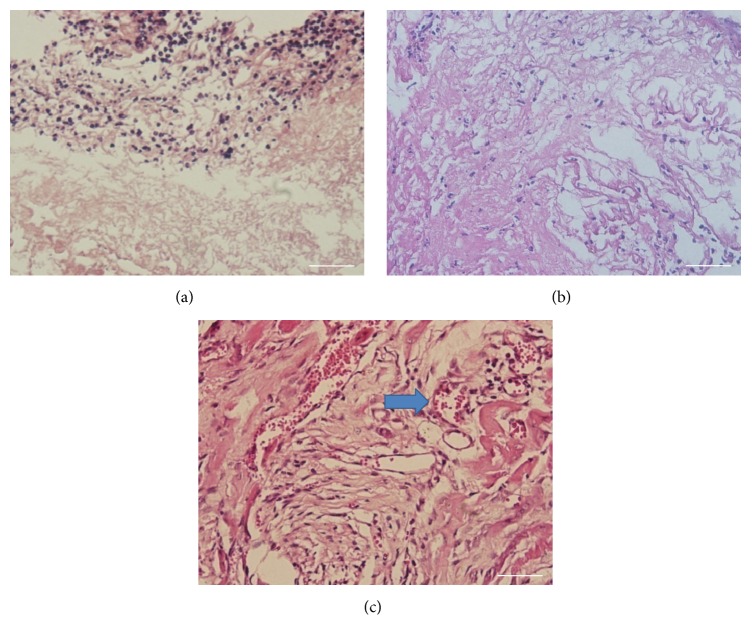
Biocompatibility test after in vivo implantation: (a) 3d, (b) 7d, and (c) 14d. At the implantation site, active angiogenesis can be seen clearly (Figure 3(c), arrows). Bar = 100 *μ*m.

The bioscaffold was transplanted subcutaneously to evaluate the biocompatibility in a mouse model. After 3 days, we can see a lot of neutrophils surrounding the tissue (Figure 3(a)). While the neutrophils infiltrated into the tissue implanted at 7 days after operation (Figure 3(b)), after 14 days mononuclear cells began to appear. The number of neutrophils decreased to a large extent and no multinucleate giant cells existed (Figure 3(c)). Besides, at the implantation site, we can see clearly the active angiogenesis (Figure 3(c), arrows). The results confirm that the decellularized pancreatic scaffold is a biocompatible scaffold.

### 3.4. Recellularization of the Pancreatic Scaffold

**Figure 4:**
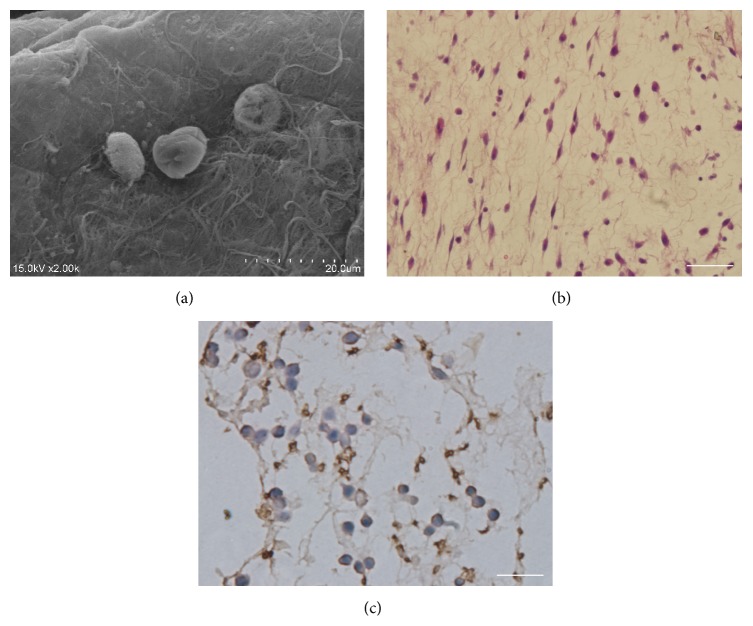
Test after recellularization: (a) SEM, (b) HE staining, and (c) IHC. Bar = 100 *μ*m.

To further verify the recellularization ability of the bioscaffold, MIN-6 cells (30 × 10^6^) were infused into the bioscaffold through the portal vein by multistep infusion technique as the previous study described [21]. Five days later, the recellularized bioscaffold was evaluated by SEM (Figure 4(a)), HE staining (Figure 4(b)) which confirmed the colonization of MIN-6 cells, and IHC (Figure 4(c)) which confirmed the normal function of the engrafted cells.

### 3.5. Insulin Gene Expression

**Figure 5:**
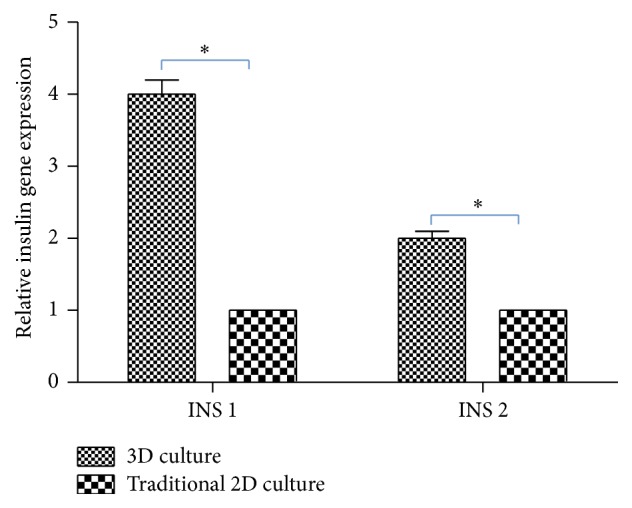
Insulin gene expression of MIN-6 seeded on 3D pancreatic ECM was higher than cells cultured on the culture dish. ^*∗*^ *P* < 0.05.

Insulin gene expression of the engrafted cells in the bioscaffold was higher than cells cultured on the culture dish. Insulin gene expression of the cells cultured on the culture dish was defined as 1 (Figure 5) (*P* < 0.05).

### 3.6. Preliminary In Vivo Functional Evaluation: Fasting Blood Glucose Test

**Figure 6:**
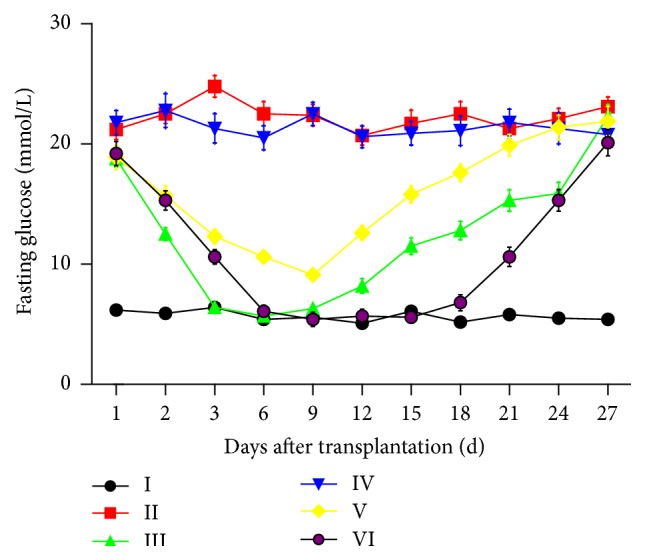
Fasting blood glucose monitoring after in vivo implantation. I: healthy mice; II: diabetic mice without interference; III: diabetic mice with subcapsular injection of the liver of MIN-6 *β* (cell amount: 2 × 10^5^); IV: diabetic mice with scaffold implantation group; V: diabetic mice with recellularized scaffold implantation group (cell amount: 2 × 10^5^); VI: diabetic mice with recellularized scaffold cubes implantation group.

After peritoneal injection of STZ, fasting blood glucose of the average mice was maintained at above 20 mM, which suggested the successful establishment of diabetic model in mice. Blood glucose of the six groups was tested for 27 days. As Figure 6 showed, differences can be seen between each group. In group III, the blood glucose fell to normal on the 3rd day. But from the 9th day on it began to rise. In group V, the blood glucose fell from the 1st day but did not descend to normal. After 9 days, it began to rise. In group VI, the falling rate was slower than the 3rd group. And fasting blood glucose fell to normal at the 6th day. After 18 days, it began to rise gradually.

## 4. Discussion

The manufacture of decellularized pancreatic matrix by means of perfusion offers another method for tissue engineering of the pancreas. The demands for a successful decellularization protocol are (1) removal of nearly all the cellular material and (2) conservation of ECM components as well as an intact vascular tree [26].

Firstly, the decellularized pancreas that we made met the first demand: no nuclear material left [27]. This requirement is of great importance because even a few nuclear materials in the bioscaffold may lead to serious cytocompatibility issues in vitro and adverse immunological response in vivo [28–30]. HE staining and SEM analysis demonstrated that the decellularization protocol can effectively remove most of the cells while still preserving the architecture of the native pancreas. DNA assessment confirmed the very small amount of remaining nuclear material quantificationally at the same time. Compared to the previous study concerning decellularized pancreatic matrix, we introduced a new physical method which is called freeze-thaw cycle. According to the literature, freeze-thaw cycle can dissolve cells in tissue and organ effectively and thus facilitate the later decellularization protocol, while having minor influence on ultramicrostructure of the tissue or organ [22]. Besides, freezing and thawing cycle can reduce immunogenicity of the bioscaffold such as leukocyte infiltration [23].

An intact preservation of ECM composition and vascular structure is as important. Immunofluorescence staining of collagen I illustrated the retaining of important extracellular matrix of decellularized pancreatic matrix compared to the native pancreas. The result of blue dye injection showed the slow flow from large blood vessel to the small vessels without any visible leakage. Intact vasculature can serve as a route through which nutrition and oxygen are transported to the engrafted cells.

Our ultimate goal is to create an organ which is suitable for transplantation. So the issue of biocompatibility must be taken into consideration. Subcutaneous implantation of the decellularized pancreas and its histological assessment indicated that the bioscaffold was biocompatible and it had certain potential of inducing angiogenesis, the biocompatibility and potential of inducing angiogenesis contributing the possibility of success of in vivo study afterwards.

After recellularization of the bioscaffold, HE staining and SEM effectively proved the colonization of the seeded cells. IHC and qRT-PCR, respectively, verified the normal function of MIN-6 cells morphologically and genetically.

Furthermore, in vivo plantation of the recellularized bioscaffold showed its potential of controlling blood glucose. However, we did observe the gradually increasing blood glucose, which indicated that the seeded cells might die due to the lack of continuous supply of nutrition. To further prove our assumption, group VI was introduced into the experiment, from which we can draw a conclusion that it was the lack of continuous supply of nutrition that led to the cell death rather than the bioscaffold itself.

Therefore, we assume that decellularized pancreas can serve as an ideal platform for the study of pancreatic bioengineering. It has a 3D structure which is essential for the survival and normal function of the cells seeded. Successful recellularization of MIN-6 cells is our first step. As a matter of fact, cell resource to repopulate the 3D bioscaffold is MIN-6 cell. Because MIN-6 cell is a kind of tumor cell, its significance is limited [21]. Our next step is to seed the scaffold with the *β*-like cells that we have already made [24]. They are autologous, and there is no need to worry about immune response. We know that an intact vasculature is preserved after the decellularization process, so we suppose that to anastomose the portal vein of the decellularized pancreatic scaffold and the vessel of the diabetic mouse may mimic the circulatory system in vivo; thus a stabilized blood supply is obtained. It will no doubt push forward the pancreatic tissue engineering in a big step.
